# A novel *MAPT* variant (E342K) as a cause of familial progressive supranuclear palsy

**DOI:** 10.3389/fneur.2024.1372507

**Published:** 2024-04-19

**Authors:** Hang Li, Qijun Li, Qin Weng, Ruixue Cui, Tzu-Chen Yen, Yanfeng Li

**Affiliations:** ^1^Department of Neurology, Peking Union Medical College Hospital, Chinese Academy of Medical Sciences and Peking Union Medical College, Beijing, China; ^2^Department of Nuclear Medicine, Peking Union Medical College Hospital Chinese Academy of Medical Sciences and Peking Union Medical College, Beijing, China; ^3^Beijing Key Laboratory of Molecular Targeted Diagnosis and Therapy in Nuclear Medicine, Beijing, China; ^4^School of Medical and Life Sciences, Chengdu University of Traditional Chinese Medicine, Chengdu, China; ^5^APRINOIA Therapeutics Co., Ltd., Suzhou, China

**Keywords:** Chinese pedigree, *MAPT* gene, progressive supranuclear palsy, E342K, ^18^F-florzolo-tau-PET

## Abstract

**Background:**

*MAPT* variants are a known cause of frontotemporal dementia and Parkinsonian syndrome, of which progressive supranuclear palsy syndrome (PSP) is a rare manifestation.

**Objective:**

To report a novel *MAPT* variant in a PSP pedigree with autosomal dominant inheritance pattern, and to produce a literature review of PSP patients with *MAPT* variants.

**Methods:**

A comprehensive clinical, genetic, and molecular neuroimaging investigation was conducted on a 61 years-old female proband diagnosed with PSP. We also collected the clinical presentation data and history of the patient’s pedigree, and performed further genetic analysis of 4 relatives, from two generations, with and without symptoms.

**Results:**

The proband exhibited typical clinical manifestation of PSP. A cranial MRI revealed midbrain atrophy, and an FDG-PET scan suggested hypo-metabolic changes in caudate nucleus, left prefrontal lobe, both temporal poles, and midbrain. ^18^F-florzolo-tau-PET revealed tau-protein deposits in the thalamus and brainstem bilaterally. A gene test by whole-exome sequencing identified a novel *MAPT* variant [NM_005910.6, exon 11, c.1024G > A (p.E342K)], and the same variant was also identified in one affected relative and one asymptomatic relative, a probable pre-symptomatic carrier.

**Conclusion:**

The PSP pedigree caused by the novel *MAPT* (E342K) variant, expanded the mutational spectrum of *MAPT*.

## 1 Introduction

Progressive supranuclear palsy (PSP), a rare neurodegenerative disorder of unknown origin, is pathologically characterized by abnormal tau deposition in the form of globose neuro-fibrillary tangles, tufted astrocytes, coiled bodies, and threads, with a predominance of 4-repeat (4R) tau isoforms (1, 2). Clinically, PSP is heterogeneous and may manifest various phenotypes, in which the most classical form is Richardson Syndrome (PSP-RS, also known as Steele-Richardson-Olszewski Syndrome), with symptoms such as vertical supranuclear gaze palsy, postural instability, and motor and cognitive deficits. Other forms of PSP have been increasingly recognized, including PSP with predominant parkinsonism (3), PSP with progressive gait freezing (4), PSP with predominant corticobasal syndrome (5), PSP with a predominant speech/language disorder (6), PSP with predominant frontal presentation (7), PSP with predominant cerebellar ataxia (5), and PSP with mixed pathology (8). Although PSP is generally considered sporadic, an increasing number of familial cases have drawn attention to its potential genetic basis (8–10). Among these, variants in the *MAPT* gene are most commonly associated with PSP (11, 12).

We identified a PSP family with an autosomal inheritance pattern, performed an exhaustive clinical, neuroimaging investigation of the proband, and further performed gene analysis of the proband and four relatives with or without PSP symptoms for the purpose of confirming the pathogenicity of potential gene variant. Additionally, we reviewed the literature on *MAPT* variants and their related disease.

## 2 Materials and methods

### 2.1 Ethics, consent, and permissions

All steps of the investigation, including approval for genetic testing, were approved by the Ethics Committee of the Peking Union Medical College Hospital. Written informed consent was obtained from subjects under investigation.

### 2.2 Clinical and family history investigations

The proband underwent a thorough clinical and familial history assessment, along with a neurological examination. A neuropsychologist administered a standardized battery of cognitive tests. The medical records of affected family members, both alive and deceased, made available by other centers, underwent a thorough review.

### 2.3 Neuroimaging

#### 2.3.1 Magnetic resonance imaging

MRI examinations were performed using a 3-T MRI scanner (Avanto, Siemens, Erlangen Germany). Axial, sagittal, and frontal T1-weighted (TE 2.7, TR 6.4), T2-weighted (TE 85.0, TR 4611.0), and fluid attenuated inversion recovery (FLAIR) (TE min full, TR 1745.0) were performed for analysis. TR, repetition time; TE, time echo; time in milliseconds.

#### 2.3.2 ^18^F-FDG positron emission tomography and ^18^F-florzolo-tau-PET scan

The ^18^F-FDG tracer was prepared at the Department of Nuclear Medicine, Peking Union Medical College Hospital, and had a radiochemical purity of over 95%. Before the administration of ^18^F-FDG, patients were required to fast for a minimum of 6 h and maintain a blood glucose concentration <7 mmol/L. The tracer dosage was set at 3.7 MBq/kg, which was administered intravenously. After the injection, patients were advised to rest in a quiet, dimly lit environment for approximately 60 min. Then, PET/CT imaging was conducted using either a PoleStar m680 (SinoUnion Healthcare, Beijing, China) or a Siemens (Erlangen, Germany) scanner. A low-dose CT scan was used for attenuation correction, followed by a 10 min brain PET 3D scan. PET images were then reconstructed using the ordered subset expectation maximization method.

The other tracer used in the investigation (^18^F-florzolotau) was prepared by the PET Center of the Department of Nuclear Medicine, Peking Union Medical College Hospital, with an injection dose of 370 MBq, time to collect after injection (90 min), and collecting time of 20 min. The patient remained with eyes closed in a quiet, light-avoidance environment after static injection of the tracer. After the tracer was injected, the brain images were acquired using a PET/CT machine (Polestar m680) in a quiet, light-protected environment, with the patent’s eyes closed, for about 90 min. A low-dose head CT scan was used for attenuation correction; a 20 min three-dimensional (3D) PET scan of the brain was performed. PET-image reconstruction was performed using the ordered subsets expectation maximization (OSEM) method.

### 2.4 Genetics

Blood samples were collected from the proband (III-9) and from the other four family members (III-5, III-7, IV-9, IV-10). Whole-exome sequencing was conducted using standard procedures, with genomic DNA extracted from peripheral blood and purification carried out using established protocols. The GenCap Whole Exome Capture Kit from MyGenostics GenCap Enrichment Technologies was utilized for library preparation, and sequencing was performed on DNBSEQ-T7. After sequencing, the raw data were saved in FASTQ format and subjected to bioinformatic analysis. Initial steps involved filtering out sequencing adapters and low-quality reads (<80 bp) using Cutadapt. After quality control, the clean reads were mapped to the UCSC hg19 human reference genome through the Burrows–Wheeler Aligner (BWA), achieving an average sequencing depth exceeding 100×. The targeted region coverage was more than 20× for over 90% and 10× for over 95%. Picard tools were employed to remove duplicated reads, and the mapping reads were utilized for variation detection. Subsequently, Genome Analysis Toolkit (GATK) Haplotype Caller was used to detect SNP and InDel variants, followed by variant filtration using GATK Variant Filtration. The resulting data were transformed into VCF format. For further annotation, variants were associated with multiple databases such as gnomAD, 1,000 Genomes, dbSNP. Predictions were made using SIFT, PolyPhen-2, mutationTaster, GERP++, phyloP, and phastCons. Sequence-variation interpretation adhered to the American College of Medical Genetics and Genomics (ACMG) guidelines. The candidate’s variant sites were confirmed by Sanger sequencing of all the members of the family. The target sequences were among those sequenced on an ABl3730 analyzer (Applied Biosystem). Sites of variation were identified by comparing DNA sequences with the corresponding GenBank reference sequences using mutation Survey software.

### 2.5 Literature review

We conducted a literature review by searching the PubMed database, with the latest search date being December 1, 2023. The keywords used included “tau,” “*MAPT*,” and “microtubule-associated protein tau,” each paired with “PSP” in various combinations. We included all patients who had been diagnosed with PSP or PSP-like conditions with *MAPT* variant.

## 3 Results

### 3.1 Case report (Proband, III-9)

Patient III-9 was a 61 year-old right-handed female farmer. Five years ago, she began to have the symptoms as unexplained recurrent falls, reduced stride, and unsteady walking, which worsen with time. After 2 years, she began to gradually develop more symptoms such as choking while drinking water, limited mouth opening, and excessive salivation. For the past 6 months, she has been completely dependent on others for daily activities, walking, and urinary incontinence. Immediate memory decline, hand tremors, and mood changes such as irritability and emotional lability, were also noted. During neurological examination, the proband was in a wheelchair and had flat facial expressions, decreased, hesitant speech output, but no comprehension difficulty. She exhibited restricted vertical gaze on pursuit and saccades, notable bradykinesia with difficulty getting out of the chair, and a tendency to fall backwards. She had normal muscle strength and notable rigidity of her neck and limbs with bilateral pyramidal signs. She struggled with finger-to-nose and heel-to-shin coordination tests.

After admission, the patient underwent neuropsychological assessment. The MMSE score was 20/30, and her MoCA score was 17/30 (specific scores are as follows: Visuospatial/Executive-1, Naming-2, Attention-5, Language-2, Abstraction-1, Delayed recall-0, Orientation-5, Education-1). The patient’s Unified Parkinson’s Disease Rating Scale (UPDRS) score improved by only 13% after the levodopa challenge test (LCT). Bladder ultrasound revealed a residual volume of 17 mL post-voiding, and her prone blood pressure was stable. Cardiac MIBG imaging showed no significant sympathetic-nerve impairment.

### 3.2 Family history

**Figure 1:**
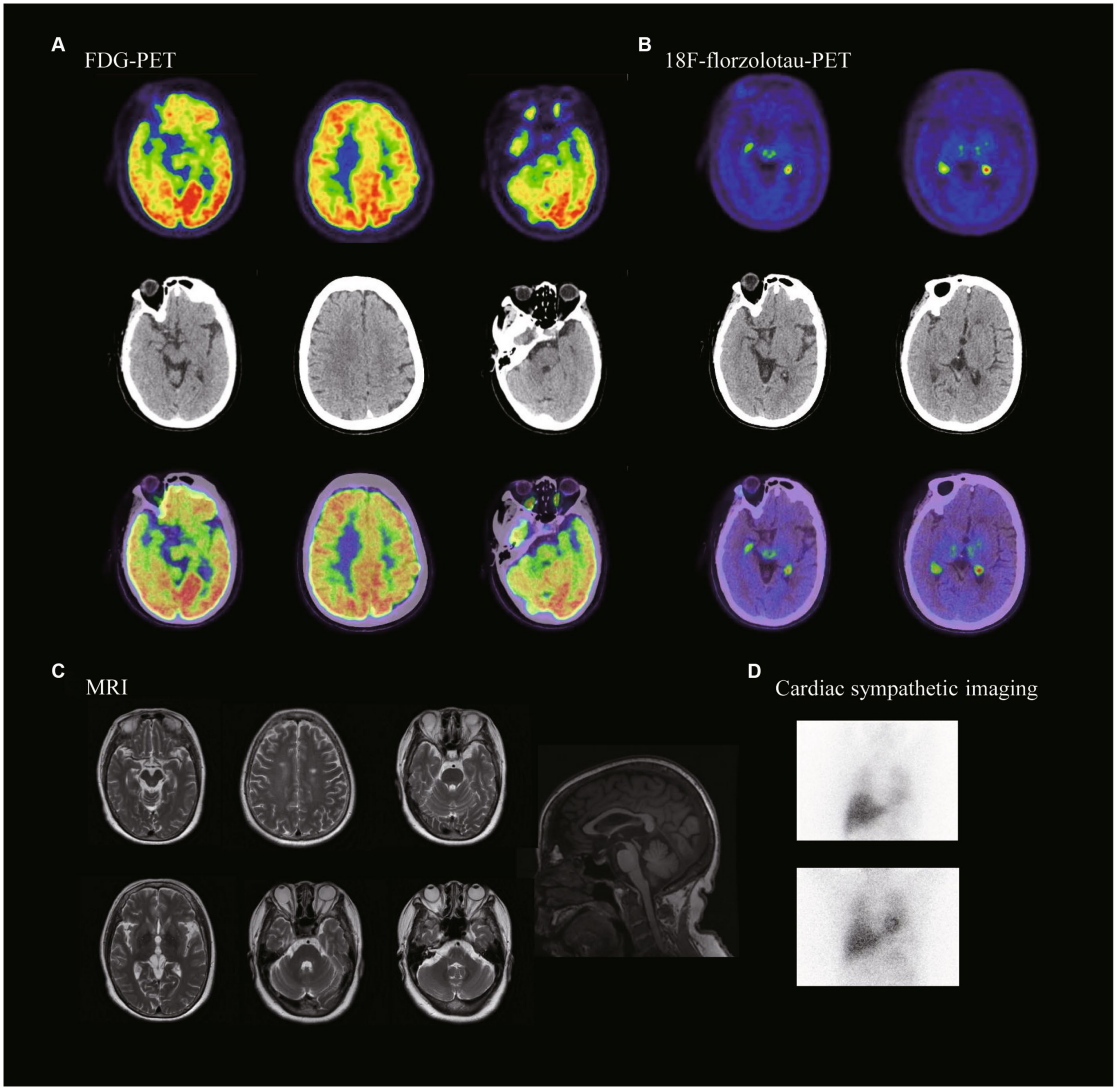
Neuroimaging of the patient with MAPT variant. **(A)** FDG-PET showed hypometabolism in the midbrain, left prefrontal lobe and bilateral temporal poles. **(B)** 18F-florzolotau-PET showed tau protein deposition seen in bilateral thalamus and brainstem. **(C)** MRI showing midbrain atrophy. **(D)** Cardiac sympathetic imaging showed no damage to cardiac sympathetic nerves. (15 min heart/mediastinum ratio (H/M ratio) = 2.56; 4 h heart/mediastinum ratio (H/M ratio) = 2.63).

The family history was significant; it showed an autosomal inheritance pattern, as shown in the pedigree depicted in Figure 1. The medical histories of all patients in her family, both alive and deceased, were reviewed, including medical records and additional information from caregivers. The age at onset of symptoms in the three deceased patients in her family was between 50 and 60 years old. All three deceased patients had shown walking slowness, easy falling frequent falls, partial gaze palsy, and memory decline. The duration of the disease of the three deceased patients was about 12–15 years.

Patient III-2 started showing postural instability at about 58 years of age and is now 71. Patient III-7 is 62 years old. She began showing amnesia at about age 53. Deceased patient I-2 presented with postural instability at about age 50 and died at age 65 when she was unable to take care of herself. Patient II-1 developed symptoms of postural instability at about age 50 and died at age 63. Patient II-3 developed symptoms of postural instability at about age 55 and died at age 67. Individual IV-9 is now age 32 with no abnormalities on neurologic examination or neuropsychological evaluation.

The clinical manifestations of this pedigree for family members and genetic analysis results are shown in Table 1.

**Table 1:** The clinical manifestations of this pedigree.

	III-9	III-2	III-7	I-2	II-1	II-3
Variant	E342K c.1024G > A	NA	E342K c.1024G > A	NA	NA	NA
Gender	Female	Male	Female	Male	Male	Male
Age onset (yr)	56	58	53	50	50	55
Age at death (yr)				65	63	67
Initial symptom(s)	Postural instability	Postural instability	Amnesia	Postural instability	Postural instability	Postural instability
Clinical symptoms	Until 5 yr. after onset	Until 13 yr. after onset	Until 9 yr. after onset			
Parkinsonism						
Bradykinesia	+	+	+	+	+	+
Rigidity	+	+	+	+	+	+
Tremor	−	−	−	−	−	−
Postural instability	+	+	+	+	+	+
Response to dopaminergic therapy	−	NA	−	NA	NA	−
Personality changes	−	−	+	+	+	−
Supranuclear gaze palsy	+	+	+	−	−	+
Optokinetic nystagmus	−	−	−	NA	−	−
Eyelid opening apraxia	−	−	−	NA	NA	−
Pyramidal signs	+	+	+	NA	NA	NA
Dysarthria	+	−	+	NA	NA	NA
Amnesia	+	+	+	+	NA	+
Perseverative vocalizations	+	+	+	+	+	+
Urinary incontinence	+	−	−	−	−	NA
Cognitive dysfunction	+	+	+	+	+	NA
MMSE	20/30	23/30	23/30	NA	NA	NA
MoCA	17/30	18/30	19/30	NA	NA	NA

NA, not available.

### 3.3 Neuroimaging

**Figure 2:**
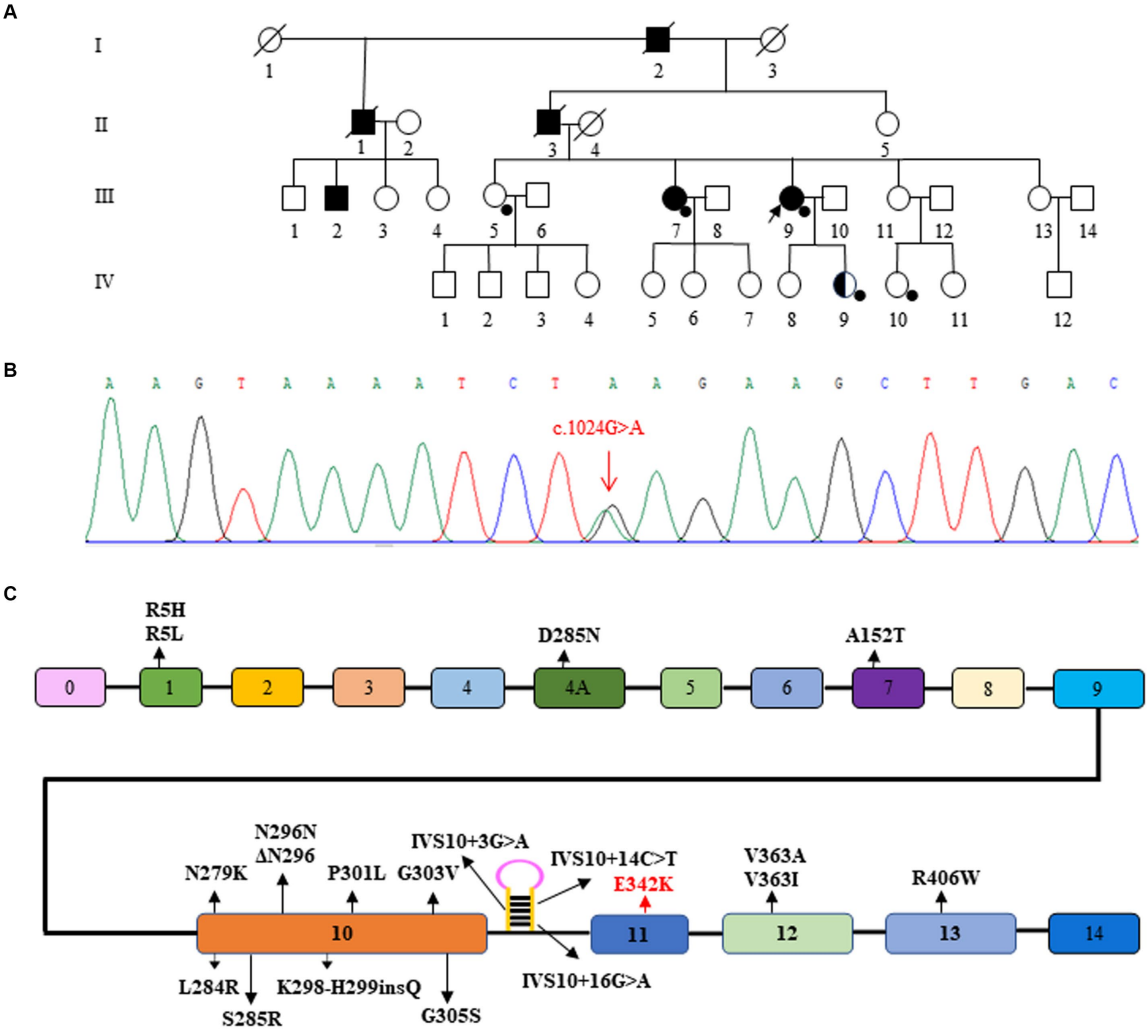
Pedigree and gene variant of the family. **(A)** The proband is indicated (arrow). Squares indicate males; circles, females; filled symbols, affected subjects; half-filled symbols, variant carriers without any clinical symptoms; open symbols, subjects without any clinical symptoms and without genetic testing, black spots, subjects with genetic testing; diagonal line through a symbol, death. **(B)** Sanger sequence showing the variant of MAPT (p. E342K, c.1024G > A). **(C)** Schematic representation of the MAPT gene showing the sites of the variants reported so far. The c.1024G > A variant in this Chinese family is highlighted in red.

An MRI of the proband’s head indicated midbrain short-axis/pontine short-axis = 0.40, and MRPI = (pontine area/midbrain area) × (midcerebellar peduncle/supracerebellar peduncle) = 11.17. PET/CT scans suggested reduced metabolism in the caudate nucleus bilaterally, left prefrontal lobe, both temporal poles and midbrain. ^18^F-florzolo-tau-PET revealed tau deposition in the bilateral thalamus and brainstem (Figure 2). Based on most widely used diagnostic criteria (8), she was diagnosed with PSP-RS.

### 3.4 Genetic analysis

A novel variant E342 [NM_005910.6, exon 11, c.1024G > A (p.E342K)] located in exon 11 of *MAPT*, which was detected in the proband (III-9), III-7, and IV-9. IV-9, the proband’s second daughter, could be an non-symptomatic carrier. III-5 and IV-10, as healthy controls, did not carry the variant. According to the Standards and Guidelines for the Interpretation of Sequence Variants, the variant is considered likely pathogenic.

### 3.5 Review of PSP-relevant cases with MAPT variant

There were 27 analyzed studies on *MAPT* variants in 40 PSP cases. These cases of clinically or neuro-pathologically diagnosed PSP or PSP-like conditions (Table 2) (13–39) had *MAPT* variants primarily in exon 10 and its stem-loop region. Of these, 28 patients (70%) had nine types of variants in exon 10, and 4 patients (10%) had three types of variants in stem-loop of exon 10. The geographic distribution of these cases was as follows: 18 cases from Japan, with a total of 6 different variants; and 20 cases from Western countries, with a total of 14 different variants. With regard to the demographics and clinical features, about 86.5% cases had a family background of neurodegenerative diseases, and there were more males (64.9%) than females. The mean onset age was 45.6 (SD = 7.95) years (range = 36–75 years), the disease duration was 7.8 (SD = 6.66) years (range = 1–27 years). It seemed that patients with the ΔN296 had an earlier age at onset (in their thirties), and two patients with R406W showed longer disease course (26 and 27 years). Broad heterogeneity of *MAPT* variants, predominantly in exon 10 and its stem-loop region, was revealed in these studies. Although there was regional variability in the types of variants observed, the age at onset seemed consistent across geographical boundaries. Notably, those with a family history of neurodegenerative conditions were more commonly affected.

**Table 2:** MAPT variants causing PSP-like syndrome.

Mutation Region	Variant Type	Author, year	Country	Clinical Phenotypes	Onset age	Sex	Death age	Course of disease	Family history	Clinical diagnosis	Pathological diagnosis
Ex1	R5H	Hayashi (13)	Japan	Amnesia and disorientation	75	M	81	6	Yes	FTDP-17	PSP-like
	R5L	Poorkaj (14)	America	Gait disorder, postural instability with falling, dysarthria, and micrographia	62	F	67	5	No	PSP	PSP
Ex4A	D285N	Higgins (15)	America	NA	NA	NA	NA	NA	NA	PSP	NA
Ex7	A152T	Coppola (16)	America	NA	NA	NA	NA	NA	NA	PSP	tauopathy
Ex10	N279K	Yasuda (17)	Japan	Bradykinesia, tremor	41	M	49	8	Yes	PNLD	PSP-like
		Delisle (18)	France	Mental slowness, indifference, memory and attention	40	M	47	7	Yes	FTDP-17	NA
			France	Indifference, attention disturbances	38	M	NA	NA	Yes	FTDP-17	NA
		Soliveri (19)	Italy	Personality changes	47	F	NA	NA	Yes	PSP	NA
		Ogaki (20) Ogaki (21)	Japan	Oscillopsia, micrographia, shuffling gait	42	M	54	12	Yes	PSP	PSP-like
			Japan	Clumsiness, oscillopsia	44	M	56	12	Yes	PSP	NA
			Japan	Parkinsonism	46	M	NA	NA	Yes	PSP	NA
			Japan	Shuffling gait and bradykinesia	41	F	51	10	No	PSP	NA
			Japan	Walking difficulty	42	F	54	12	Yes	PSP	NA
			Japan	Unstable gait and character changes and memory problems	43	F	51	8	Yes	PSP	NA
		Oka (22)	Japan	Bradykinesia and tremor	40	M	NA	NA	Yes	PSP	NA
			Japan	Trouble concentrating and bradykinesia	42	M	46	4	Yes	bvFTD	PSP
	L284R	Rohrer (23)	England	Personality changes, backward falls	43	F	47	4	Yes	PSP	NA
		Cullinane (24)	England	Difficulty focusing on near objects and unsteadiness	41	M	45	4	Yes	PSP	CBD
	S285R	Ogaki (20)	Japan	Speech and breathing disorder	46	M	49	3	No	PSP	NA
		Fujioka (25)	Japan	Dystonia and supranuclear gaze palsy	40	M	44	4	Yes	PSP	PSP-AD
			Japan	Bradykinesia	41	M	44	3	Yes	PSP	PSP
	ΔN296	Pastor (26)	Spain	Memory disorder, word-finding problems and slowness	38	M	NA	NA	Yes	Atypical PSP	NA
		Rossi (27)	Italy	Falls, dysarthria; slowing of ocular movements	36	M	NA	NA	Yes	PSP-like	NA
	N296N	Ogaki (20)	Japan	Parkinsonism	44	M	NA	NA	Yes	PSP	NA
	K298-H299insQ	Nakayama (28)	Japan	Neck stiffness, postural instability	60	M	NA	NA	Yes	PSP	NA
			Japan	Gait disturbance cognitive dysfunction	NA	F	NA	NA	Yes	PSP	NA
			Japan	Gait difficulty cognitive dysfunction	NA	M	NA	NA	NA	NA	NA
	P301L	Bird (29)	America	Tremor, speech impairment	41	M	46	5	Yes	APD	PSP-like
		Kaat (30)	Netherlands	NA	NA	NA	NA	NA	Yes	PSP	NA
	G303V	Ros (31)	Spain	Parkinsonism, falls, micrographia, dysarthria, ocular motor damage	37	M	NA	NA	Yes	PSP	PSP
	S305S	Stanford (32)	Australia	Dystonia, dysarthria, falls, bradykinesia	48	F	51	3	Yes	PSP	PSP
		Rodríguez (33)	Columbia	Behavioral changes and abnormal movements	53	M	57	4	Yes	PSP	NA
IVS10	IVS10 + 3G > A	Spina (34)	America	Dizziness, neck rigidity	47	M	48	1	Yes	Atypical PSP	tauopathy
			America	Dizziness, neck rigidity	52	F	58	6	Yes	Atypical PSP	PSP-like
	IVS10 + 14C > T	Omoto (35)	Japan	Clumsiness, tremor, appetite loss, apathy	44	F	NA	NA	Yes	Perry syndrome	PSP-like
	IVS10 + 16G > A	Morris (36)	England	Fatigue, micrographia, character changes	40	M	45	5	Yes	PSP	Tauopathy
Ex12	V363I	Parmera (37)	Brazilian	Gait disorder, visuospatial difficulties, rigidity, and dexterity	47	F	NA	NA	No	PSP-CBS and PCA	NA
	V363A	Rossi (38)	Italy	Diplopia, falls, bradykinesia	53	M	NA	NA	Yes	PSP	NA
Ex13	R406W	Ygland (39)	Sweden	Dyscalculia, social withdrawal, apathy	50	F	76	26	Yes	AD	PSP-like
			Sweden	Personality change	53	F	80	27	YES	AD	PSP-like

NA, not available; PSP, progressive supranuclear palsy; PNLD, pallido-nigro-luysian degeneration; FTDP-17, frontotemporal dementia and parkinsonism linked to chromosome 17; AD, Alzheimer’s disease; CBS, corticobasal syndrome; CBD, corticobasal degeneration; APD, atypical Parkinson’s disease; PCA, posterior cortical atrophy; PSP-AD, PSP with concomitant AD; PSP-CBS, PSP with concomitant CBS. “PSP-like” pathology means that tau accumulations exist in brain but do not meet the pathological diagnostic criteria of PSP.

A summary of clinical manifestations in PSP patients with *MAPT* gene variants suggested an insidious onset in all cases, with varying clinical manifestations. The first symptoms of the 37 cases studied, listed according to frequency, were as follows: bradykinesia (13/37); postural instability/gait disturbance (10/37); personality changes (10/37); cognitive dysfunction (7/37); tremor (6/37); dysarthria (5/37); supranuclear gaze palsy (4/37); amnesia (4/37); neck rigidity (3/37); general rigidity (3/37); and dizziness (2/37).

## 4 Discussion

We examined a newly identified Chinese family with a novel *MAPT* variant, displaying typical PSP features. Those features included recurrent falls, oculomotor challenges, and progressive cognitive impairment with short-term memory loss. Additionally, a literature review of previously reported *MAPT*-mutated PSP patients demonstrated the heterogeneity and complexity of the disease caused by different genetic mutations, which has broadened our knowledge of *MAPT* variants and disease with tau-pathology.

The *MAPT* gene, situated on chromosome 17q21, encodes the tau protein and comprises 16 exons. Exons 0 and 14 do not contribute to protein encoding. Inheritance is autosomal dominant (40, 41). Over 100 families worldwide have been identified with 50 distinct variants in the tau gene. Tau proteins exist in six isoforms, resulting from selective splicing in exons 2, 3, and 10. Depending on the splicing of exons 2 and 3, isoforms with 0, 1, or 2 N-terminal repeats (0 N, 1 N, 2 N) are produced. Selective splicing of exon 10 results in tau isoforms with either 3 or 4 microtubule-binding repeats (40). Disorders involving *MAPT* are primarily characterized by frontotemporal lobe dementia and/or clinical Parkinson’s syndrome, and less commonly by motor neuron disease (42). In case-control genome-wide association studies (GWAS), *MAPT* is the most significant risk locus for sporadic PSP, and is also the most common cause of familial PSP (11, 12).

The novel *MAPT* variant is deemed likely pathogenic based on criteria set forth by the American College of Medical Genetics and Genomics (ACMG) and the Society for Molecular Pathology (43). These criteria are as follows: the mutation resides in a functional structural domain (PM1). It is absent in healthy populations (PM2). The new missense alteration occurs at an amino acid residue (E342V) previously identified as pathogenic (PM5) (44, 45). The mutation co-segregates with the disease in multiple family lines (PP1). The affected gene is highly conserved across species, and computational analyses suggest the mutation is deleterious (PP3). Clinical presentation of patients supports a monogenic etiology for the disease (PP4).

The variant is situated in the fourth repeat, R4, of *MAPT*’s exon 11, which encodes the functional microtubule-binding domain (MTBD). Spanning from the latter part of exon 9 to exon 12 (amino acids 244–368), the MTBD contains four highly conserved 18-amino-acid repeats, separated by either 13 or 14 residues. MTBD plays a critical role in the interaction of tau with microtubules, promoting their assembly and stability (46, 47). The p.E342V variant in exon 11 has demonstrated an increase in 4R tau mRNA levels. This variant not only escalates the splicing of exon 10 but also decreases the inclusion of exons 2 and 3. At both RNA and protein levels, the E342V variant hampers the ability of tau to promote microtubule assembly. Additionally, the variant is hypothesized to diminish the affinity of tau for microtubules, thereby facilitating its release, self-assembly, and fibrillar aggregation (45). Pathological examination has unveiled notable frontotemporal lobe neuronal loss, intracytoplasmic tau aggregates, and paired helical tau filaments, thus confirming the variant’s deleterious effects. Moreover, exon 11 appears to influence the alternative splicing of other exons in the tau gene (44).

The *MAPT* gene is the most frequently implicated gene in hereditary forms of PSP. As shown in Table 2 and Figure 1, which contain variants in this patient’s genes, there were 20 different *MAPT* variants described in cases presenting with PSP (clinical or neuropathologic diagnosis). Variant prevalence varies regionally, potentially due to ethnic and environmental influences. The occurrence of *MAPT* variants in PSP patients ranges from 0.6 to 14.3% (25, 48–50). The most commonly observed *MAPT* variant in PSP is at codon 279, found in 10 patients. Families carrying the N296 variant tend to experience an earlier onset of the disease. Almost all patients with *MAPT* variants are heterozygous, with only one reported case of a homozygous N296 variant. That particular patient exhibited a more severe phenotype and disease progression than did those with heterozygous variants (23). The p.K298H299insQ variant in exon 10 represents the first reported insertion variant in the *MAPT* gene. Patients with *MAPT* variants typically exhibit a gradual disease onset and are more likely to present with Parkinsonian symptoms. Pathologically, these patients often manifest standard PSP or PSP-like conditions. Table 2 shows that the vast majority of patients with *MAPT* variants, with the exceptions of R5L, V363I, and V363A, have a family history of Parkinsonian syndrome, dementia, or other neurodegenerative diseases. This supports the previously published notion of familial clustering in PSP (27, 51). Clinical heterogeneity has been observed even among patients carrying the same variant, including those within the same family, underscoring the complex interaction of genetic and environmental factors in PSP (33, 34, 37).

In the family we studied, the mean age at onset was 53.7 years. This is higher than the mean age at onset in the general PSP population with *MAPT* variants, which is approximately 45.8 years. The family’s mean duration of the disease was 13.3 years. This is longer than the mean disease duration in the general PSP population with *MAPT* variants, which is approximately 8.4 years. The older age at onset and longer disease duration in this family could suggest the presence of family-specific genetic or environmental factors that modulate the course of the disease. Also of interest is the presence of a genetic variant in IV-9 without clinical manifestations, which may be related to the fact that the patient was very young at the time of the present study. Future follow-up of this patient is needed to further understand this disease.

The predominance of postural instability as the initial symptom in this family aligns with observations from the broader PSP population with *MAPT* variants. This could have implications for early diagnosis and intervention strategies. The presence of amnesia as an initial symptom in one individual emphasizes clinical heterogeneity, even within families carrying the same *MAPT* variants. Memory disorder is seldom found to be one of the main symptoms of PSP, but has been described in association with *MAPT* variants in PSP (Table 2). The differences in age at onset and disease duration between this family and the general PSP population call for more in-depth genetic and environmental studies to uncover the reasons behind these discrepancies.

The hummingbird and morning-glory-flower signs on MRI, previously established as markers of midbrain pathology, effectively distinguish patients with PSP from those with Parkinson’s disease and multiple-system atrophy. The hummingbird sign has a 99.5% diagnostic specificity, and the morning-glory sign a 97.7% diagnostic specificity for PSP. As depicted in Figure 2 (52), our patient exhibited pronounced hummingbird and morning-glory signs. The patient’s MRI also revealed a notably small midbrain, which is indicative of a classic PSP-RS phenotype. This significant midbrain atrophy aligns well with the clinical manifestations commonly observed in patients with sporadic PSP-RS. In summary, the patient’s MRI findings are consistent with established literature, reinforcing their diagnosis as a case of PSP-RS (53–55).

Studies have indicated that ^18^F-Fluorodeoxyglucose PET often reveals hypometabolism in specific regions like the midbrain, basal ganglia, thalamus, and frontal lobes in patients with PSP-RS (56, 57).The most frequently observed sites of hypometabolism are the thalamus (100%), caudate nucleus (86%), midbrain (86%), and frontal lobes (71%) (58). In line with these findings, our patient also exhibited hypometabolism in these regions, as well as in the bilateral temporal poles. Uniquely, our patient exhibited hypometabolism in the bilateral temporal poles. Kanel et al. posited that a greater loss of cholinergic activity in the superior temporal poles could explain this observation in patients with PSP (59).

Although structural MRI did not reveal significant atrophy in our patient, the functional deficits were evident. Prior research has identified focal, bilateral cortical thinning in patients with PSP, predominantly in the prefrontal/precentral cortex and the temporal pole. Even in the absence of structurally significant changes in our case, the patient did present with clinically relevant cognitive deficits (60). These additional findings contribute to the complexity of the PSP clinical picture. The hypometabolism in the bilateral temporal poles could serve as another layer of diagnostic criteria, potentially distinguishing subsets of PSP patients or highlighting disease severity. Moreover, although the absence of significant cortical thinning may limit the utility of structural MRI in some PSP cases, functional markers such as hypometabolism can provide valuable insights into the disease process, especially when aligned with clinical manifestations like cognitive deficits.

Our proband demonstrated tau protein deposition in the bilateral thalamus and brainstem. The most commonly used tau tracer, ^18^F-AV-1451, typically shows increased uptake in multiple brain regions in PSP-RS, including the pallidum, chiasma, caudate nucleus, and thalamus (61, 62). Previous research has consistently noted increased ^18^F-AV-1451 uptake in the globus pallidus, putamen, and caudate nucleus, among other regions, when comparing PSP-RS with controls. Quantitative analyses have found that the best differentiation between PSP-RS and control groups is achieved through evaluating globus pallidus retention and thalamic activity (63, 64). Our proband’s tau-PET results, which showed tau protein deposits in the bilateral thalamus and brainstem, align with previous studies (65). These imaging markers further substantiate the diagnosis of PSP and contribute to our understanding of this disorder.

## 5 Limitations

The key constraints of our study include the limited size of our cohort and the relatively few documented cases of PSP and PSP-like conditions with *MAPT* variants in existing literature. Such limitations could introduce publication or selection bias, as well as inconsistencies in symptom reporting and grading across different studies. To fully grasp the complex clinical heterogeneity observed in patients with *MAPT* variants, larger sample sizes are needed.

Ethical constraints also prevented further genetic studies among other family members to clarify the gene’s pathogenicity, though we plan to pursue this in future research. Additionally, the review may be subject to publication bias, particularly because genetic screening is not commonly performed in some rural areas of China. To affirm the prevalence of *MAPT* variants in China, broader multicenter genetic studies are necessary.

## 6 Conclusion

The PSP pedigree caused by the novel *MAPT* (E342K) variant have been expanding the mutational spectrum of *MAPT*. The findings in the present study may be helpful for the development of targeted therapeutic interventions of PSP.

## Data availability statement

The raw sequence data reported in this paper have been deposited in the Genome Sequence Archive (Genomics, Proteomics & Bioinformatics 2021) in National Genomics Data Center (Nucleic Acids Res 2022), China National Center for Bioinformation/Beijing Institute of Genomics, Chinese Academy of Sciences (GSA-Human: HRA006913) that are publicly accessible at https://ngdc.cncb.ac.cn/gsa-human.

## Ethics statement

This study was approved by the ethical review board of Peking Union Medical College Hospital. The studies were conducted in accordance with the local legislation and institutional requirements. The participants provided their written informed consent to participate in this study. Written informed consent was obtained from the individual(s) for the publication of any potentially identifiable images or data included in this article.

## Author contributions

HL: Writing – original draft, Writing – review & editing, Data curation, Investigation. QL: Writing – original draft, Writing – review & editing. QW: Writing – original draft, Writing – review & editing, Software. RC: Writing – original draft, Writing – review & editing. T-CY: Writing – original draft, Writing – review & editing, Resources. YL: Writing – original draft, Writing – review & editing, Funding acquisition, Project administration, Resources, Supervision.
